# Treatment with morning blue light increases left amygdala volume and sleep duration among individuals with posttraumatic stress disorder

**DOI:** 10.3389/fnbeh.2022.910239

**Published:** 2022-09-12

**Authors:** William D. S. Killgore, John R. Vanuk, Natalie S. Dailey

**Affiliations:** Department of Psychiatry, College of Medicine, University of Arizona, Tucson, AZ, United States

**Keywords:** PTSD, blue light treatment, sleep, voxel based morphometry (VBM), neuroimaging (anatomic)

## Abstract

**Background:**

Posttraumatic stress disorder (PTSD) is associated with numerous cognitive, affective, and psychophysiological outcomes, including problems with sleep and circadian rhythms. We tested the effectiveness of a daily morning blue-light exposure treatment (BLT) versus a matched amber light treatment (ALT) to regulate sleep in individuals diagnosed with PTSD. Moreover, PTSD is also associated with reliable findings on structural neuroimaging scans, including reduced amygdala volumes and other differences in cortical gray matter volume (GMV) that may be indicative of underlying neurobehavioral dysfunctions. We examined the effect of BLT versus ALT on GMV and its association with sleep outcomes.

**Methods:**

Seventy-six individuals (25 male; 51 female) meeting DSM-V criteria for PTSD (*Age* = 31.45 years, *SD* = 8.83) completed sleep assessments and structural neuroimaging scans, followed by random assignment one of two light groups, including BLT (469 nm; *n* = 39) or placebo ALT (578 nm; *n* = 37) light therapy daily for 30-min over 6-weeks. Participants wore a wrist actigraph for the duration of the study. After treatment, participants returned to complete sleep assessments and a structural neuroimaging scan. Neuroimaging data were analyzed using the Computational Anatomy Toolbox (CAT12) and Voxel-Based Morphometry (VBM) modules within the Statistical Parametric Mapping (SPM12) software.

**Results:**

The BLT condition produced significant increases in total time in bed and total sleep time from actigraphy compared to the ALT condition, while ALT improved wake after sleep onset and sleep efficiency compared to BLT. Additionally, BLT led to an increase in left amygdala volume compared to ALT but did not affect hypothesized medial prefrontal regions. Finally, within group correlations showed that improvements in sleep quality and nightmare severity were correlated with increases in left amygdala volume over the course of treatment for the BLT group but not the ALT group.

**Conclusion:**

In individuals with PTSD, daily exposure to morning blue light treatment was associated with improvements in objective sleep duration and increased volume of the left amygdala compared to amber placebo light treatment, and changes in amygdala volume correlated with subjective improvement in sleep. These findings suggest that daily morning BLT may provide an important non-pharmacologic adjunctive approach for facilitating sleep and neurobehavioral recovery from PTSD.

## Introduction

Exposure to a traumatic event can produce significant cognitive, emotional, and physiological changes within an individual. When severe and persistent, these changes may lead to stress-related outcomes, such as posttraumatic stress disorder (PTSD), a condition characterized by frequent re-experiencing symptoms, avoidance of situations or thoughts related to the trauma, negative mood, cognitive alterations, and increased arousal and reactivity (American-Psychiatric-Association, 2013). These recurrent symptoms are emotionally distressing and often interfere with normal social and occupational functioning. While most traumatic experiences do not lead to PTSD, the disorder remains prevalent and will affect up to 7% of the general population at some point over their lifespan (Kessler et al., 2005). Moreover, the prevalence of PTSD is even greater among certain groups such as combat Veterans (Hoge et al., 2004; Tanielian and Jaycox, 2008) who are at increased risk of exposure to traumatic events.

Posttraumatic stress disorder has also been associated with structural and functional changes within the brain. While the neurophysiological response to traumatic stress is complex and affects myriad systems throughout the central nervous system, several brain regions have been consistently identified as particularly relevant to the etiology and maintenance of PTSD, including the amygdala, medial prefrontal cortex, and hippocampus (Bremner, 2006; Liberzon and Martis, 2006). Among these, the amygdala, a brain region associated with the evaluation of affective valence and emotional salience, has often emerged as one of the most consistently implicated brain structures in the neuropathology of PTSD. Early functional magnetic resonance imaging (fMRI) studies repeatedly demonstrated that PTSD is often characterized by hyperresponsive activation of the amygdala to emotional or trauma-related stimuli (Rauch et al., 1996, 2000; Liberzon et al., 1999; Shin et al., 2004, 2005). Furthermore, the exaggerated amygdala responses have often been found to co-occur with reduced activation within areas of the medial prefrontal cortex, such as the anterior cingulate, subcallosal cortex, and medial orbitofrontal regions (Liberzon et al., 1999; Shin et al., 2004, 2005; Bremner et al., 2005), brain regions believed to be critical to emotional evaluation and regulation. Not only do these brain regions show significant functional differences between patients with PTSD and healthy controls, but these regions also appear to show corresponding differences in gray matter volume (GMV). While there is some inconsistency across studies, the most common findings includes reduced GMV in several structures including the amygdala (Morey et al., 2012; Starcevic et al., 2014; Logue et al., 2018; Ousdal et al., 2020), medial prefrontal cortex, and hippocampus (Franz et al., 2020; Del Casale et al., 2022). Decreased volume of the amygdala appears to be a particularly common finding, but there is uncertainty as to whether this represents a stable pre-existing risk factor for PTSD or if volume reductions emerge as a result of the stress response to trauma. Moreover, there is ample evidence to suggest that GMV is somewhat plastic and can be modified with experience (Ditye et al., 2013; Sun et al., 2016; Wenger et al., 2017, 2021; Ueno et al., 2018) or treatment (Perini et al., 2017; Butler et al., 2018; Mancke et al., 2018; Husain et al., 2019; Wang et al., 2019; Brancati et al., 2021; Schading et al., 2021; Soshi et al., 2021; Yang et al., 2021). Nevertheless, there has been relatively little research examining longitudinal changes in GMV over a course of treatment or with measured changes in relevant PTSD symptoms. The present study will attempt to address this gap by evaluating GMV changes in PTSD and their association with sleep outcomes.

In the present study, we focus on sleep disruption as it is one of the most common symptoms of PTSD. Sleep disruption is so closely linked with PTSD that it is sometimes called the “hallmark” symptom of the disorder, with prevalence rates for sleep problems ranging from 70% to 91% across studies. Among those with PTSD, self-reported sleep quality is often described as poor and fragmented (Van Liempt, 2012) and often includes problems falling asleep, staying asleep, early morning awakening, and frequent and severe nightmares (Neylan et al., 1998; Ohayon and Shapiro, 2000). Insomnia in the period prior to a traumatic event has been shown to increase vulnerability to developing PTSD (Gehrman et al., 2013). Similarly, sleep disruption, particularly rapid eye movement (REM) sleep, in the acute period following a trauma has also been associated with the development of PTSD (Mellman et al., 2002). Sleep is critical for healthy social and emotional functioning (Goldstein and Walker, 2014; Ben Simon et al., 2020). When people lack sleep, emotional stability is adversely affected (Kahn-Greene et al., 2007; Killgore et al., 2008). Neuroimaging research has shown that sleep deprivation weakens the functional connectivity between the medial prefrontal cortex regions involved in emotional regulation and the emotionally reactive amygdala, leading to hyperresponsiveness of the amygdala to emotional stimuli (Yoo et al., 2007). One theoretical model proposes that REM sleep provides the ideal neurochemical balance of adrenergic to cholinergic activity in cortical modules to allow the brain to strip away unhealthy affective tone from memories, allowing them to be reconsolidated at a more manageable emotional intensity over time (Walker and Van Der Helm, 2009). According to this model, the sleep disruption common to PTSD may impair this normal affective balancing process, preventing full recovery. The important role of sleep in healthy emotional functioning and its disruption in PTSD has prompted many researchers to propose a primary focus of treatment toward sleep problems as a vehicle for bringing about effective recovery from the disorder (Gilbert et al., 2015; Miller et al., 2019).

The most common approaches for treating sleep disorders in people with PTSD include pharmacotherapy or cognitive behavioral therapy for insomnia (CBT-I) (Weber and Wetter, 2021). While treatment with hypnotic medications or benzodiazepines can initially facilitate a soporific state, there are many health and performance related drawbacks to the regular use of pharmacologic treatments for insomnia, and patients and their providers are often dissatisfied with the treatment (Hermes et al., 2013). Studies have suggested improvements in sleep with CBT-I for this population, but the majority still do not achieve full remission of insomnia (Talbot et al., 2014). Consequently, further research into alternative approaches is warranted.

Circadian disruption is common following exposure to a trauma and has even been proposed as a “core feature” of trauma-related disorders (Agorastos and Olff, 2020). Consequently, optimizing the underlying circadian rhythm is another potential approach that could be applied to facilitate sleep in people with PTSD. The human propensity for sleep is directly linked with the diurnal circadian rhythm of melatonin secretion by the pineal gland. For humans, the most efficient and restorative sleep occurs when the individual’s sleep/wake pattern closely aligned with the circadian day and night (Lavie, 2001; Arendt, 2006). The circadian rhythm of sleep and melatonin production is most powerfully affected by ocular exposure to light (Cajochen et al., 2003). Because the retina contains melanopsin-based intrinsically photosensitive retinal ganglion cells (ipRGCs) that are primarily stimulated by blue-wavelength light, even brief periods of blue light exposure can significantly affect melatonin and the timing of the circadian rhythm (Provencio et al., 2000; Panda et al., 2005; Qiu et al., 2005). Morning exposure to blue-wavelength light has the effect of phase advancing the rhythm (i.e., shifting the sleep period earlier in the evening), while light in the evening will produce a phase delay (i.e., shifting the sleep period later into the night). Accordingly, we have successfully applied morning blue light treatment (BLT) to improve the sleep and circadian functioning of individuals with mild traumatic brain injury (mTBI) (Killgore et al., 2020; Raikes et al., 2021). Using a simple light-box device for 30-min each morning, we were able to shift the circadian rhythm of sleep/wake, improve cognitive performance, and influence brain structure and functional connectivity in these individuals (Bajaj et al., 2017, 2021; Killgore et al., 2020; Raikes et al., 2020, 2021). Two recent studies have examined the potential for bright light exposure treatment in PTSD, with promising preliminary outcomes suggesting reductions in symptoms (Zalta et al., 2019; Youngstedt et al., 2021). However, those studies used either bright white light or a weaker green light than in our prior work, and findings did not focus on sleep or brain structure. So, it remains to be determined whether sleep problems associated with PTSD could be modified by blue light and whether this would relate directly to changes in critical brain structures commonly implicated in the disorder.

For the present study, we conducted a 6-week clinical trial using the same light intensity and wavelength as in our prior mTBI studies described above. Participants completed a baseline assessment of subjective and objective sleep followed by 6-weeks of daily morning BLT or an identical treatment with an amber light treatment (ALT) as a placebo control, and a final post-treatment assessment of sleep. Moreover, each assessment also included a structural neuroimaging scan to assess gray matter volume (GMV) in segmented regions of the brain. Based on prior research described above, we hypothesized that BLT would increase objective sleep duration and subjective sleep quality relative to ALT and that these improvements would correlate with increased GMV of the amygdala and medial prefrontal cortex.

## Materials and methods

### Participants

**FIGURE 1:**
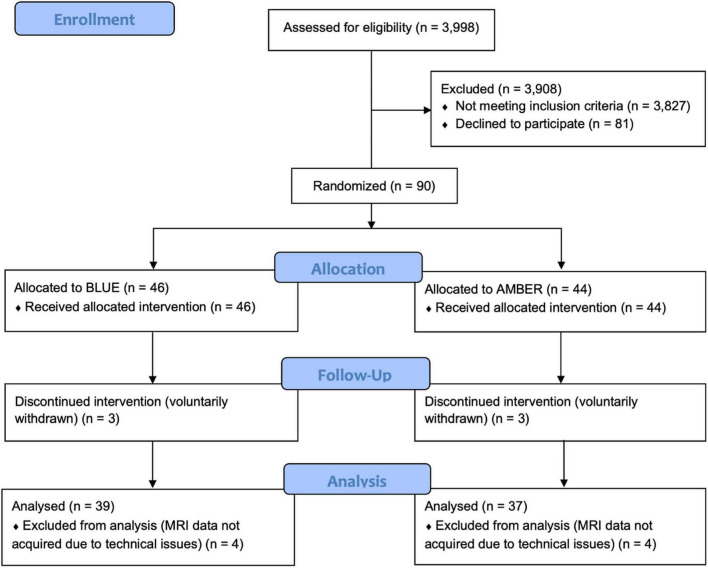
CONSORT diagram showing the assignment of participants to each condition. Following an intention-to-treat approach, all participants who were randomly assigned to a treatment condition were analyzed, with the exception of participants who withdrew from the trial prematurely (*n* = 3 per condition) or did not have usable neuroimaging scans at both sessions (*n* = 4) per condition.

A total of 90 individuals meeting DSM-V criteria for PTSD were initially recruited and randomized to one of the treatment conditions (30 male; 60 female; 47 BLT; 23 ALT) with an average age of 31.09 years (*SD* = 8.72). As shown in Figure 1, participant drop-out and missing data reduced the final complete dataset by 14 participants. Thus, for the complete dataset, a total of 76 individuals (25 male; 51 female) completed the light exposure treatment and underwent pre- and post-treatment assessments and structural neuroimaging scans. Participants in the complete dataset ranged from 20 to 49 years of age (*M* = 31.45, *SD* = 8.83). These participants self-identified as White (61.8%), Hispanic/Latino (22.4%), Black/African American (3.9%), Native-American/American Indian (2.6%), Asian/Pacific Islander (1.3%), Other (7.9%), and there were no significant differences in the racial/ethnic breakdown between light conditions, χ^2^(*df* = 5) = 4.03, *p* = 0.545. Most participants indicated that they had used marijuana at some point in their lifetime, but this did not differ between groups [BLT: 71.8%; ALT: 83.8%, χ^2^(*df* = 1) = 1.57, *p* = 0.21]. Furthermore, most participants reported using marijuana fewer than 100 times during their lifetime, with no differences between groups (BLT: 58.1%; ALT: 57.2%). A total of 28% of those in the BLT group reported using marijuana in the month prior to their baseline session, while 16.1% of those in the ALT had done so, but this difference was not significant, χ^2^(*df* = 1) = 1.33, *p* = 0.25, Demographic breakdown for the two conditions is provided in Table 1.

**TABLE 1:** Baseline demographic characteristics.

Baseline demographics	Blue (active) *n* = 39		Amber (placebo) *n* = 37		*P*-value
	M	SD	M	SD	
Male/Female (n)	14/25	–	11/26	–	0.63
Age	32.08	9.01	30.78	8.72	0.53
Education	14.00	1.86	13.71	1.67	0.49
Full Scale IQ	100.08	10.22	102.97	13.65	0.30
Age at Index Trauma	28.33	8.65	27.58	8.30	0.70
Years since Index Trauma	3.38	2.36	3.12	2.23	0.60
Baseline PCL-5	48.38	12.97	44.51	12.48	0.19
Baseline CAPS-5 Number of Symptoms	13.33	2.68	12.35	3.42	0.17
Baseline CAPS-5 Symptom Severity	35.41	8.12	32.57	10.09	0.18
% With Direct Trauma Exposure	89.7%	–	78.4%	–	0.17
Trauma Type (within light group)					0.27
Physical Abuse/Assault	41.0%	–	32.4%	–	
Sexual Abuse/Assault	20.5%	–	27.0%	–	
Accident (e.g., car, work-related)	10.3%	–	2.7%	–	
Natural Disaster	2.6%	–	2.7%	–	
War/Combat	17.9%	–	10.8%	–	
Other	7.7%	–	24.3%	–	
PSQI Total	10.06	2.90	9.38	3.43	0.35
DDNSI Total	13.84	5.38	14.66	4.82	0.49
ISI Total	15.28	4.61	14.41	6.11	0.48

Participants were recruited from the local Tucson and surrounding metropolitan areas *via* posted flyers, radio advertisements, and internet campaigns. Interested individuals completed a telephone screening interview to determine initial eligibility. Those meeting eligibility requirements were invited to complete an in-person laboratory assessment session (i.e., Visit 1) that included a Structured Clinical Interview for DSM-V (SCID-V) administered by a trained researcher. Selected participants were required to meet criteria for PTSD and be between the ages of 18 and 50 years, right-handed according to the Edinburgh Handedness Inventory (Oldfield, 1971), and be a primary English speaker. Exclusionary criteria included any history of head injury with loss of consciousness exceeding 30 min or post-traumatic amnesia lasting longer than 24 h. Individuals were also excluded if they had any history of neurological illness, chronic medical condition, or comorbid psychiatric condition (excluding depression), or an index trauma event occurring before the age of 18 years or an index trauma that occurred longer than 10 years prior to the current study. Potential participants were also excluded if there was evidence of ongoing trauma (e.g., currently in an abusive relationship), or an index trauma that was considered to be non-qualifying based on current diagnostic standards (e.g., emotional/verbal abuse, exposure to natural deaths due to age or illness). Volunteers were also excluded for abnormal visual acuity that could not be corrected with contact lenses, prior history of light treatment, history of light induced conditions (e.g., epilepsy, migraine) or other medical conditions that could be affected by light, measured intelligence below 70, metal in the body, pregnancy, or other contraindications for MRI, and use of medications that might affect neuroimaging outcomes (e.g., beta-blockers, mood stabilizers, etc.), current or upcoming use of sleep-inducing medications (e.g., zolpidem) or sleep altering supplements (e.g., melatonin), evidence of lower than 6th grade reading comprehension, or use of illicit substances (although marijuana was permitted). A total of 90 participants were enrolled, however, eight participants failed to complete the study and six other participants did not have complete neuroimaging scans due to technical issues. Figure 1 presents the CONSORT flow diagram.

All interested individuals were briefed on the study procedures and requirements and provided written informed consent prior to enrollment. Study procedures were evaluated and approved by the Institutional Review Board of the University of Arizona College of Medicine and the United States Army Human Research Protections Office.

### Procedure

Over a 7-week protocol, each participant attended three visits to the laboratory, including an intake (Visit 1), baseline assessment and scan (Visit 2), and post-treatment assessment and scan (Visit 3). After Visit 2, participants were randomly assigned to complete a daily half-hour morning light exposure regimen of either blue light treatment (BLT) or amber light treatment (ALT) for 6 weeks.

#### Visit 1: intake

The intake visit involved completion of the informed consent process and administration of the SCID by a trained research technician. Individuals meeting criteria for PTSD were fitted with a wrist actigraph device to assess sleep and wake over the course of the study (see below).

#### Visit 2: baseline assessment and MRI scan

Following 1 week of baseline sleep assessment, participants returned to the lab to complete a baseline cognitive assessment and a series of neuroimaging scans. Participants completed an extensive battery of assessments, some of which are discussed in other publications. The present article focuses on subjective sleep assessments and actigraphy measures and their association with structural neuroimaging findings. Neuroimaging scans were initiated at 9:00 a.m. Daily assessment activities were highly scheduled throughout the day. Upon completion of the assessments and scans, each participant received a light exposure device (described below). The use of the device was demonstrated to the participant, and they were provided with a printed instruction brochure that provided detailed information about its use.

#### Six-week light therapy period

Based on a computer-generated randomization scheme, participants were randomly assigned to receive either a BLT or ALT light device. Treatment was administered double-blind (i.e., participants were not informed that there were different colors of lights being used and all study staff with direct participant contact were blind to the color of the light device assigned). Participants were instructed to use the assigned device every morning for 30-min, within 2 h of awakening, but no later than 11:00 a.m. This was done to ensure that the light exposure occurred within time-window generally associated with phase advancement of the circadian rhythm, while still allowing some flexibility of use within a naturalistic home setting. At each treatment session, the lightbox was set at approximately arm’s length (20–30 in. from the face) at a slight angle (20–40°), so that both sides of the face would be exposed to the light. To minimize visual discomfort, participants were encouraged to refrain from looking directly at the light panel. The device automatically deactivated after 30 min of continuous use. Participants were also instructed to log their light use and previous night’s sleep each morning *via* a secure online portal.

#### Visit 3: post-treatment assessment and MRI scan

After 6 weeks of daily treatment with morning light exposure, participants returned to the lab for a final assessment session (Visit 3). This final session involved essentially the same assessments and procedures as the baseline visit (Visit 2). Upon completion, all equipment was returned and participants were released from the study.

### Equipment and assessment measures

#### Light exposure devices

Each participant was provided with a small light therapy device, fitted with either blue or amber light emitting diodes (LEDs). The devices were manufactured by Philips Electronics (Stamford, CT, United States), and were identical in design, except for the color wavelength emitted by the LEDs. The devices consisted of a 13.5 × 14 cm plastic-encased flat box that was table-mounted and included a 10 × 6 panel array of LEDs. A commercially available Philips goLITE BLU^®^ Energy Light device (Model HF3321/60) was used for the active BLT condition. This device has a narrow bandwidth [peaking at λ = 469 nm, at 214 Lux, and single panel irradiance (mW/cm^2^) = 0.11 at 80 cm]. The ALT condition was provided by an identical appearing device that was fitted LEDs that emitted amber wavelength light [peaking at λ = 578 nm, at 188 Lux, and panel irradiance (mW/cm^2^) = 0.04 at 80 cm]. Amber light was selected as a control condition as prior research has used it successfully as a placebo (Raikes and Killgore, 2018), and evidence suggests that it has a significantly lower effect on melatonin phase shifting (Geerdink et al., 2016), and brain connectivity (Killgore et al., 2022), than blue light, but still provides a plausible and believable light condition. Participants were required to log into a secure website each day to record the time when the light was used.

#### Posttraumatic stress disorder assessments

The Structured Clinical Interview for DSM-V (SCID) (First, 2015) was administered at Visit 1 to ensure individuals met diagnostic criteria for a current PTSD diagnosis. For those who were enrolled in the study, the Clinician Administered PTSD Scale for DSM-5 (CAPS-5) (Weathers et al., 2018) was administered at Visit 2 and Visit 3 to assess PTSD total symptom severity and the number of symptoms meeting threshold.

#### Subjective sleep assessments

Participants completed several subjective sleep assessments, including the Pittsburg Sleep Quality Index (PSQI), a measure of sleep habits and sleep quality in the past month (Buysse et al., 1989), the Disturbing Dreams and Nightmares Severity Index (DDNSI), a measure of the frequency and severity of nightmares (Krakow et al., 2002), and the Insomnia Severity Index (ISI), a measure of both nighttime and daytime insomnia components (Bastien et al., 2001).

#### Objective sleep assessments

Throughout the course of the study, participants wore an Actiwatch Spectrum Pro^®^ (Philips Respironics, Bend, Oregon) on their dominant hand. Sleep was scored using the automated algorithms of the Actiware 6^®^ program with further hand editing to ensure accuracy compared to sleep diaries. For the present analysis, we averaged the minutes of sleep obtained for each overnight sleep opportunity for the first six nights of the baseline week (i.e., between Visit 1 and 2) and the final six nights preceding the post-treatment visit (Visit 3). Actigraphic data were first scored for all sleep during each 24-h period (i.e., including nap periods), and again when only nocturnal sleep was included (i.e., excluding daytime naps). For the present report, we only used the nocturnal sleep scores. These included standard scores for total Time in Bed (TIB; the total amount of time spend in the rest period from bed-time to rise time), Total Sleep Time (TST; the total number of minutes scored as sleep during the rest period once the individual had fallen asleep), Sleep Onset Latency (SOL; the number of minutes between the initiation of the rest period and first scored minute of sleep), Wake After Sleep Onset (WASO; the number of minutes of wake during the rest period scored after the first minute of scored sleep), and Sleep Efficiency (SE; defined as TST divided by TIB).

#### Structural neuroimaging

Structural magnetic resonance imaging (MRI) data were collected at 3T (Siemens Skyra) using a 32-channel head coil. Head movement was restricted using foam cushions during all image acquisition. Anatomical data were acquired with a high-resolution T1-weighted weighted 3D magnetization-prepared rapid acquisition gradient echo (MPRAGE) sequence (TR/TE/flip angle = 2,100 ms, 2.33 ms, 12°) comprising 176 axial slices (256 × 256 matrix) with a slice thickness of 1 mm and voxel size of 1 mm × 1 mm × 1 mm.

### Statistical analysis

As this study reflects the first of its type examining the effects of a 6-week trial of BLT versus placebo ALT on sleep and recovery from PTSD, we present findings from two analytic approaches, including the per-protocol analysis (i.e., analysis of all participants who completed the study with valid data), as well as a more conservative intention-to-treat analysis (i.e., analysis of all participants who were randomized to a treatment condition, regardless of completion status or data availability). Because some participants who were initially randomized did not complete the study and there was also some loss of data due to technical difficulties in a few cases (see Figure 1), we replaced missing data in the intention-to-treat analysis with a multiple imputation approach. The multiple imputation analysis was implemented with 5 iterations using the Missing Values module in SPSS 28 and the pooled mean was imputed as the final value for missing scores. Imputed values were calculated for the following variables: PSQI Baseline Bedtime (3 missing values), PSQI Total Baseline (5 missing values), DDNSI Total Baseline (5 missing values), ISI Total Post-treatment (7 missing values), PSQI Total Post-treatment (8 missing values), Actigraphy TIB Baseline (9 missing values), Actigraphy TST Baseline (9 missing values), Actigraphy SOL Baseline (9 missing values), Actigraphy WASO Baseline (9 missing values), Actigraphy SE Baseline (9 missing values), Actigraphy TIB Post-treatment (11 missing values), Actigraphy TST Post-treatment (11 missing values), Actigraphy SOL Post-treatment (11 missing values), Actigraphy WASO Post-treatment (11 missing values), Actigraphy SE Post-treatment (11 missing values), Intracranial Volume Mean (14 missing values), Left Amygdala Volume Baseline (14 missing values), Left Amygdala Volume Post-treatment (14 missing values), DDNSI Total Post-treatment (15 missing values).

#### Sleep outcomes

Each sleep metric was scored according to standard published criteria. The effects of light condition on each sleep metric were tested using a 2 (light condition) × 2 (baseline versus post-treatment) mixed analysis of covariance (ANCOVA), with age, sex, baseline bedtime, and total PTSD symptom severity from the CAPS-5 entered as covariates. Significant interactions were decomposed with Bonferroni protected *post hoc* tests (*p* < 0.05).

#### Voxel based morphometry

T1 weighted structural images were preprocessed using the Computational Anatomy Toolbox (CAT12, version 12.8)^1^ in SPM12^2^. Images were realigned to the anterior-posterior commissure axis and then segmented using the longitudinal pipeline into gray matter, white matter, and cerebrospinal fluid using CAT12. Segmented images were used to create a custom DARTEL template and then the images were normalized to the stereotaxic coordinate space of the Montreal Neurological Institute (MNI). Smoothing of normalized images was performed with an 8 mm full width at half maximum (FWHM) isotropic Gaussian kernel.

First, processed GMV data from CAT12 were analyzed in SPM12. For determining the effect of light conditions, a 2 between (BLT vs. ALT) × 2 within (baseline vs. post-treatment) mixed analysis of variance (ANOVA) was specified within SPM12 using the flexible factorial option, controlling for age, sex, and total intracranial volume. From this analysis, we focused specifically on the planned comparison between the magnitude of pre-to-post change for the BLT versus ALT conditions. Maps were initially thresholded for peak intensity at *p* < 0.001, with correction for multiple comparisons using a family wise error (FWE) cluster threshold of *p* < 0.05 (Woo et al., 2014). The statistical maps were constrained to two bilateral search territories defined by the AAL atlas (Tzourio-Mazoyer et al., 2002), including (1) bilateral amygdala, and (2) bilateral medial prefrontal cortex (i.e., gyrus rectus, medial orbitofrontal cortex, and anterior cingulate cortex).

To analyze the associations between sleep-related metrics and brain volume, we first calculated the difference score between the baseline and post-treatment sleep scores and the difference in GMV from baseline to post-treatment. Then, we ran a series of separate multiple regression analyses in SPM12 predicting change in GMV from the change in each sleep measure. In addition to the primary sleep measure difference scores, we also included covariates to control for age, sex, and total intracranial volume. The image maps were initially thresholded for peak intensity at *p* < 0.001, with a cluster threshold of *p* < 0.05, FWE corrected within each of the two search territories corresponding to the amygdala and medial prefrontal cortex, as described above. The first eigenvariate from significant clusters was extracted and plotted against the relevant sleep metric for visualization. To determine the potential moderating effects of light condition, these data were examined using a regression analysis for categorical moderators.

## Results

### Condition blinding

It was important to determine whether participants believed they were receiving the active condition or the placebo condition. Therefore, at the end of treatment, all participants indicated their perceived treatment group. In the present sample, 71.1% of the BLT participants believed they were receiving the active treatment, while 75.0% of the ALT participants believed they had received the active treatment, χ^2^(1) = 0.146, *p* = 0.702. Thus, there was no difference between groups with regard to their perception of which treatment they received.

### Treatment adherence

The treatment groups showed similar levels of treatment adherence, as determined by their valid daily logging of the times that they used the light device. One participant in each group failed to complete the daily logging of light use times. The BLT group recorded use of the light device on 40.65 days (SD = 5.30) and the ALT group recorded use of the light device on 41.57 days (SD = 7.40), which did not differ significantly between groups, *t*(86) = 0.67, *p* = 0.502.

### Light condition effects

This initial set of analyses aimed to determine the effect of BLT versus ALT on (1) subjective and objective sleep outcomes and (2) GMV within the amygdala and prefrontal regions of interest.

### Sleep outcomes

#### Pittsburg sleep quality index

Per-protocol analysis: After controlling for covariates, light condition did not significantly affect changes in PSQI Total scores, as indicated by a non-significant interaction, *F*(1,64) = 0.008, *p* = 0.929. Intention-to-treat analysis: Similarly, light condition did not influence changes in PSQI Total scores when all participants, regardless of adherence or completion status (including imputed scores for missing values), were included for the intention-to-treat analysis, *F*(1,84) = 0.018, *p* = 0.895.

#### Disturbing dreams and nightmares severity index

Per-protocol analysis: After controlling for covariates, light condition did not significantly affect changes in DDNSI scores, as indicated by a non-significant interaction, *F*(1,59) = 0.29, *p* = 0.592. Intention-to-treat analysis: Light condition also did not influence changes in DDNSI scores when all participants, regardless of adherence or completion status (including imputed scores for missing values), were included for the intention-to-treat analysis, *F*(1,84) = 2.62, *p* = 0.109.

#### ISI

Per-protocol analysis: After controlling for covariates, light condition did not significantly affect changes in ISI Total scores, as indicated by a non-significant interaction, *F*(1,66) = 0.57, *p* = 0.454. Intention-to-treat analysis: Similarly, light condition did not influence changes in ISI scores in the intention-to-treat analysis, *F*(1,84) = 0.51, *p* = 0.479.

#### Time in bed

**FIGURE 2:**
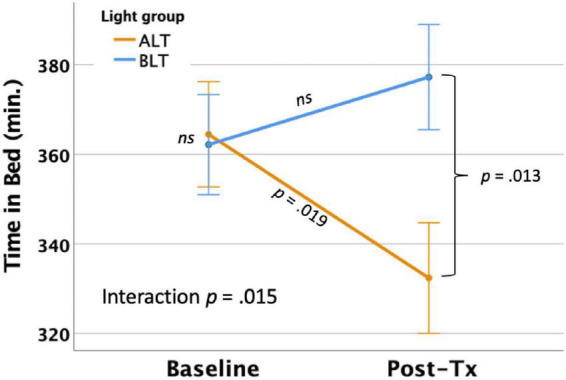
Six weeks of blue light treatment (BLT) was associated with significantly greater actigraphically measured time in bed (TIB) than an equal period of amber light treatment (ALT), *p* = 0.015. Orange line = ALT; Blue line = BLT. Error bars = ± 1 SE. *Post hoc* comparisons are Bonferroni corrected.

Per-protocol analysis: With covariates controlled, there was a significant light condition by session interaction for actigraphically measured TIB, *F*(1,59) = 6.24, *p* = 0.015 (see Figure 2), suggesting that TIB increased for BLT while decreasing for ALT. Bonferroni protected *post hoc* comparisons revealed that the interaction was primarily driven by a decline in TIB for the ALT group (*p* < 0.05), while BLT was associated with a non-significant increase in TIB over the same time period. Although groups did not differ at baseline, the BLT group showed significantly higher TIB than the ALT group after 6-weeks of treatment (*p* < 0.05). Intention-to-treat analysis: When all participants, regardless of adherence or completion status (including imputed scores for missing values), were included for the intention-to-treat analysis, the outcomes remained significant, suggesting that TIB increased for BLT while decreasing for ALT *F*(1,84) = 4.61, *p* = 0.035.

#### Total sleep time

**FIGURE 3:**
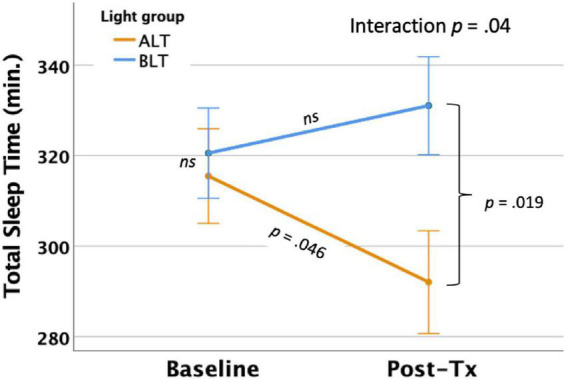
Six weeks of blue light treatment (BLT) was associated with significantly greater actigraphically measured total sleep time (TST) than an equal period of amber light treatment (ALT), *p* = 0.042. Orange line = ALT; Blue line = BLT. Error bars = ± 1 SE. *Post hoc* comparisons are Bonferroni corrected.

Per-protocol analysis: With covariates controlled, there was a significant light condition by session interaction for actigraphically measured TST, *F*(1,59) = 4.33, *p* = 0.042 (see Figure 3). Similar to the findings for TIB, Bonferroni protected *post hoc* comparisons revealed that the interaction was primarily driven by a decline in TST for the ALT group (*p* < 0.05), while BLT was associated with a non-significant increase in TST over the same time period. Although groups did not differ at baseline, the BLT group showed significantly higher TST than the ALT group after 6-weeks of treatment (*p* < 0.05). Intention-to-treat analysis: In contrast to the per-protocol analysis, when imputed data for all participants, regardless of adherence or completion status, were included for the intention-to-treat analysis, the light condition × assessment session interaction no longer reached statistical significance *F*(1,84) = 2.56, *p* = 0.114.

#### Sleep onset latency

Per-protocol analysis: After controlling for covariates, light condition did not significantly affect changes in actigraphically measured SOL Total scores, as indicated by a non-significant interaction, *F*(1,59) = 1.54, *p* = 0.219. Intention-to-treat analysis: Similarly, light condition did not influence changes in SOL scores in the intention-to-treat analysis, *F*(1,84) = 0.087, *p* = 0.768.

#### Wake after sleep onset

**FIGURE 4:**
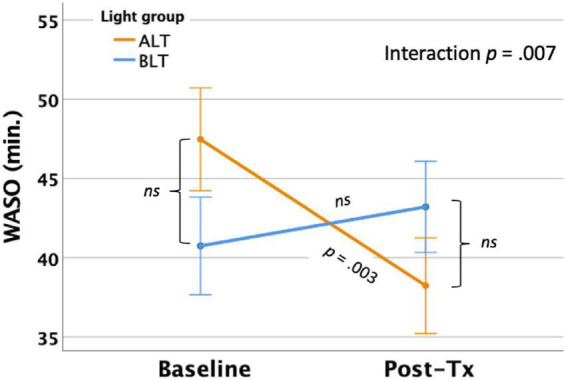
Six weeks of amber light treatment (ALT) was associated with significantly greater decrease in actigraphically measured wake after sleep onset (WASO) than an equal period of blue light treatment (BLT), *p* = 0.007. Orange line = ALT; Blue line = BLT. Error bars = ± 1 SE. *Post hoc* comparisons are Bonferroni corrected.

Per-protocol analysis: After controlling for covariates, there was a significant light condition by session interaction for actigraphically measured WASO, *F*(1,59) = 7.73, *p* = 0.007 (see Figure 4), suggesting that WASO declined significantly for the ALT group but did not change for the BLT group. Bonferroni protected *post hoc* comparisons revealed that the interaction was primarily driven by a decline in WASO for the ALT group (*p* < 0.05), while BLT was associated with a non-significant increase in WASO over the same time period. Nonetheless, groups did not differ significantly in total WASO at baseline or after 6-weeks of treatment. Intention-to-treat analysis: When all participants, regardless of adherence or completion status (including imputed scores for missing values), were included for the intention-to-treat analysis, the interaction remained significant, suggesting that WASO did not change for BLT but did decrease for ALT *F*(1,84) = 8.78, *p* = 0.004.

#### Sleep efficiency

**FIGURE 5:**
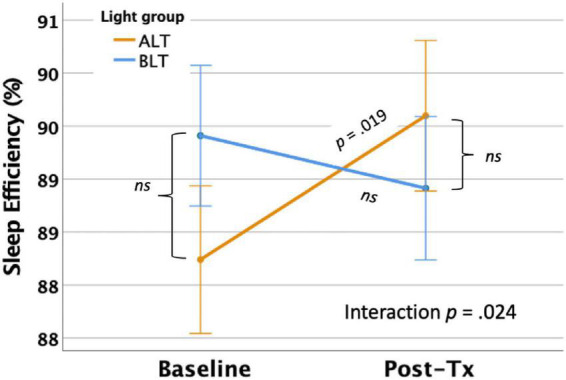
Six weeks of amber light treatment (ALT) was associated with significantly greater increase in actigraphically measured percentage sleep efficiency (SE) than an equal period of blue light treatment (BLT), *p* = 0.024. Orange line = ALT; Blue line = BLT. Error bars = ± 1 SE. *Post hoc* comparisons are Bonferroni corrected.

Per-protocol analysis: Once covariates were controlled, there was a significant interaction between light condition and session for SE as measured by actigraphy, *F*(1,59) = 5.35 *p* = 0.024 (see Figure 5). This finding suggests that overall sleep efficiency was improved for the ALT group relative to the BLT group. Bonferroni protected *post hoc* comparisons revealed that the interaction was primarily driven by an increase in SE for the ALT group (*p* < 0.05), while BLT was associated with a non-significant decline over the same time period. Groups did not differ in SE at baseline or after 6-weeks of treatment. Intention-to-treat analysis: When all participants, regardless of adherence or completion status (including imputed scores for missing values), were included for the intention-to-treat analysis, the interaction remained significant, suggesting that SE did not change for BLT but decreased for ALT *F*(1,84) = 8.12, *p* = 0.005.

### Gray matter volume

#### Amygdala

**FIGURE 6:**
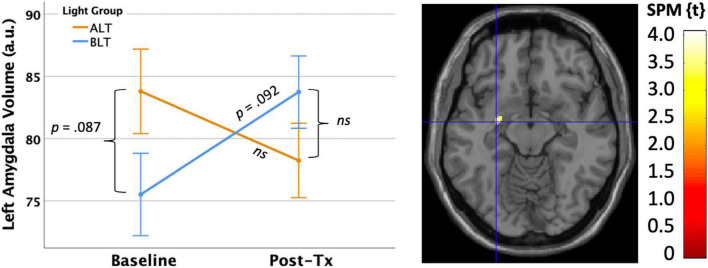
Six weeks of blue light treatment (BLT) was associated with significantly greater increase on left amygdala volume than an equal period of amber light treatment (ALT), *p* < 0.05 FWE corrected. The left side of the figure shows the mean (± SE) for amygdala volume. The right side of the figure shows the spatial location of this change [MNI: *x* = –28, *y* = –8, *z* = –12]. The color bar shows the magnitude of the t-value. Error bars = ± 1 SE. *Post hoc* comparisons are Bonferroni corrected.

First, we applied a bilateral amygdala ROI mask from the AAL atlas and compared the magnitude of pre- to post-treatment GMV change within the bilateral amygdalae for the BLT versus the ALT group, using a peak threshold at *p* < 0.001 (uncorrected) and correction for multiple comparisons at the cluster level (*p* < 0.05, FWE corrected). This analysis yielded a significant cluster (*k* = 18 voxels) in the left amygdala [MNI: *x* = −28, *y* = −8, *z* = −12] where volume increased for the BLT group relative to the ALT group over the course of treatment (see Figure 6).

#### Medial prefrontal cortex

Using the same approach described above, we also examined the effects of light condition on changes in GMV within the medial prefrontal cortex (i.e., defined by a bilateral ROI comprising the gyrus rectus, medial orbitofrontal cortex, and anterior cingulate cortex from the AAL atlas). At an initial peak threshold of *p* < 0.001 (uncorrected), with cluster correction at *p* < 0.05 (FWE), there were no regions in which GMV change was moderated by light condition over the treatment period.

### Gray matter volume correlations with sleep outcomes

Because the volume increases following 6 weeks of BLT were localized to the left amygdala, we conducted follow-up analyses within this same search territory to examine the potential associations between GMV changes and sleep-related outcomes.

#### Pittsburg sleep quality index

**FIGURE 7:**
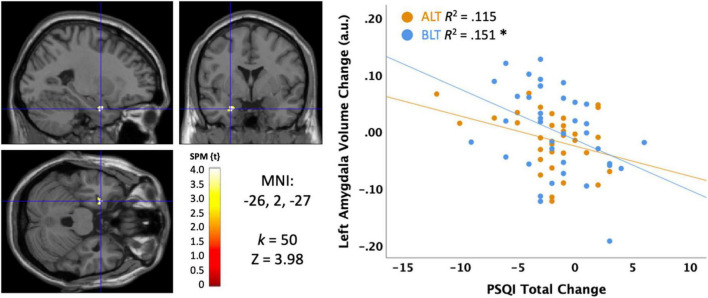
Correlation between sleep quality change on the Pittsburgh Sleep Quality Index (PSQI) and the change in left amygdala volume after 6 weeks of treatment with blue light treatment (BLT) or amber light treatment (ALT). The left panel shows the location of the significant cluster in the amygdala for the entire sample, *p* < 0.05 FWE cluster corrected. The right panel shows the scatterplot for the BLT and ALT samples separately. MNI, Montreal Neurologic Institute stereotaxic coordinates. *p < 0.05.

The primary subjective measure for sleep quality was the Total PSQI score collected at baseline and immediately after 6 weeks of light treatment. As evident in Figure 7, there was a significant negative association, suggesting that greater reductions (i.e., improvement) in PSQI scores over 6 weeks of treatment were significantly correlated with greater increases in volume within in a cluster (*k* = 50; MNI: −26, 2, −27) within the left amygdala (peak *p* < 0.001, uncorrected, cluster corrected *p* < 0.05, FWE). Because this association was for the sample as a whole, we extracted the significant voxels and assessed the influence of light condition as a moderator. However, when the interaction term for light condition × PSQI score was added to the regression, it did not account for significant variance (Change in *R*^2^ = 0.000, *p* = 0.92), suggesting that light did not moderate the effect. Nonetheless, this association was statistically significant for the BLT group (*r* = −0.388, *p* = 0.016) but showed only a trend level association for the ALT group (*r* = −0.339, *p* = 0.058).

#### Disturbing dreams and nightmares severity index

**FIGURE 8:**
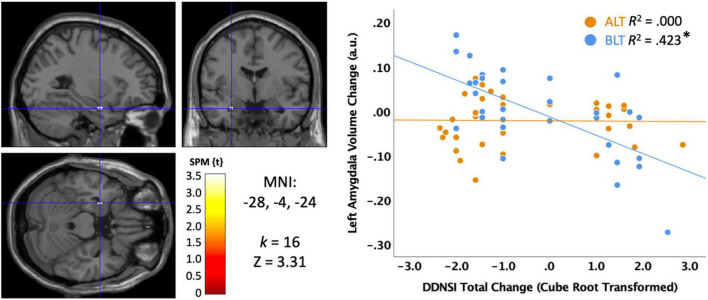
Correlation between sleep quality change on the Disturbing Dream and Nightmare Severity Index (DDNSI) and the change in left amygdala volume after 6 weeks of treatment with blue light treatment (BLT) or amber light treatment (ALT). The left panel shows the location of the significant cluster in the amygdala for the entire sample, *p* < 0.05 FWE cluster corrected. The right panel shows the scatterplot for the BLT and ALT samples separately. DDNSI change data were cube root transformed to ensure assumptions of normality. MNI, Montreal Neurologic Institute stereotaxic coordinates. *p < 0.05.

We also examined disturbing dreams and nightmare severity with the DDNSI. Initial examination of the DDNSI change data suggested a positively skewed distribution, so a cube root transformation was applied to minimize the effects of outliers. As shown in Figure 8, greater declines (i.e., improvement) in DDNSI scores over the treatment period were significantly correlated with greater volume increases in a cluster (*k* = 16; MNI: −28, −4, −24) within the left amygdala (peak *p* < 0.001, uncorrected, cluster corrected *p* < 0.05, FWE). To test moderation by light condition, we extracted the significant voxels and examined the interaction term for the light condition × DDNSI scores. This analysis suggested a trend level interaction (Change in *R*^2^ = 0.057, *p* = 0.084), but did not reach conventional levels of statistical significance. Nonetheless, the association was statistically significant for the BLT group (*r* = −0.650, *p* = 0.00004) but not for the ALT group (*r* = −0.016, *p* = 0.928). This suggests that nightmare severity may improve in concert with increases in left amygdala volume, which shows a trend toward greater increases with BLT than ALT.

#### ISI

There was no correlation between changes in ISI scores and changes in GMV within the left amygdala.

#### Time in bed

There was no correlation between changes in actigraphically measured TIB and changes in GMV within the left amygdala.

#### Total sleep time

There was no correlation between changes in actigraphically measured TST and changes in GMV within the left amygdala.

#### Sleep onset latency

There was no correlation between changes in actigraphically measured SOL and changes in GMV within the left amygdala.

#### Wake after sleep onset

There was no correlation between changes in actigraphically measured WASO and changes in GMV within the left amygdala.

#### Sleep efficiency

There was no correlation between changes in actigraphically measured SE and changes in GMV within the left amygdala.

## Discussion

Three primary findings emerged from this study of individuals with PTSD. First, when analyzed per-protocol, 6 weeks of daily morning BLT led to significantly greater objectively measured time-in-bed (TIB) and total sleep time (TST) compared to an equivalent period of ALT. This suggests that BLT was effective at modifying objective sleep parameters to improve the amount of sleep obtained in people meeting diagnostic criteria for PTSD (although the TST finding did not hold when analyzed with missing values imputed in an intent-to-treat approach). However, there was no significant effect of BLT on subjective measures of sleep outcomes regardless of the approach. Second, over the course of treatment, the BLT group showed the hypothesized increase in the volume of the left amygdala compared to the ALT group. However, the predicted increase in medial prefrontal cortex volume was not observed. Finally, we found that the observed increases in left amygdala volume over the 6 weeks of treatment were correlated with improvements in sleep quality and nightmare severity, while no associations were found for objective measures of sleep. Each of these findings are discussed in turn below.

Importantly, we found that 6 weeks of daily morning BLT led to improvements in actigraphically measured sleep (when analyzed per protocol). As shown in Figure 2, BLT was associated with about a quarter of an hour more time in bed during the last week of treatment compared to the baseline week, while those who received the placebo treatment showed the opposite pattern. Figure 3 shows that this resulted in a modest (but non-significant) increase in TST for the BLT group relative to the ALT group (who showed a significant decline over the same time frame) as well. This outcome was hypothesized based on the rationale that an earlier phase shift of melatonin onset (Cajochen et al., 2003) and regular entrainment of the circadian day would allow participants to maintain greater wakefulness during the day and fall asleep at an earlier time, which would maximize sleep by better aligning the biological night with the external environment (Lavie, 2001; Arendt, 2006). Our prior work with individuals who were recovering from an mTBI showed that BLT was effective at phase advancing the circadian rhythm of sleep by about 1 h (Killgore et al., 2020), although TST was not significantly changed. Research on the effects of light on TST has yielded mixed outcomes (Figueiro et al., 2014; Saxvig et al., 2014; Richardson et al., 2018; Wu et al., 2018) and has often been plagued by small sample sizes. Our prior mTBI study that failed to find TST effects had fewer participants than in the present PTSD study, so we had greater statistical power here to detect increases in sleep time, which may account for the differences in outcomes across studies. Further, while we found that actigraphic TST was increased by BLT relative to ALT, we did not find any effects on other objective metrics including SOL or WASO, suggesting that daily BLT did not significantly reduce the time to fall asleep or the amount of wakefulness observed after sleep onset, while this was improved for the ALT group. However, the lack of finding for WASO needs to also be considered in the context of the increased time in bed for the BLT group as well. With greater overall time in bed, there is also more opportunity for more or longer waking periods to occur, which may have contributed to the lack of improvement in WASO, despite an increase in TST. This is further supported by the lack of improvement in SE for the BLT group compared to the ALT group. Of course, wrist actigraphy is only a proxy for actual sleep and future studies would benefit from utilizing polysomnography to determine actual sleep.

In our prior studies of mTBI, we observed that BLT led to significant improvements in subjective sleep measures, such as daytime sleepiness (Killgore et al., 2020; Raikes et al., 2020, 2021) and subjective sleep quality (Raikes et al., 2020). Overall, we did not find a significant improvement in total sleep quality or nightmare severity here among those who received BLT versus ALT. When considered in light of the prior findings on objective sleep, this suggests that while individuals in the BLT condition did obtain more sleep overall, they did not self-report subjective differences in the quality of that sleep or a notable decline in the severity of nightmares. This is important and points to a potential limitation of the intervention in this group, as the subjective perception of sleep quality is an important aspect of wellbeing.

A primary objective of the current study was to examine the effect of BLT on GMV within the primary affective regulation network that has been implicated in PTSD, which includes the amygdala and medial prefrontal cortex (Rauch et al., 1996, 2000; Shin et al., 2004, 2005; Bremner et al., 2005). Prior work in PTSD samples has pointed to dysfunctional regulation of the functional responses of the amygdala by medial prefrontal regions (Rauch et al., 1996, 2000; Liberzon et al., 1999; Shin et al., 2004, 2005). This dysfunction of emotional regulation has been postulated to lead to exaggerated amygdala responses to emotional stimuli, which are remarkably similar to the prefrontal-amygdala disconnection findings that emerge during sleep deprivation (Yoo et al., 2007). When considered in light of volumetric MRI studies that have repeatedly demonstrated reduced amygdala and prefrontal volume in people with PTSD (Rogers et al., 2009; Morey et al., 2012), and evidence that amygdala volumes increase with successful treatment (Laugharne et al., 2016), we expected that our treatment with BLT would be associated with corresponding increases in amygdala (and perhaps medial prefrontal cortex) volumes. As predicted, we found that 6-weeks of BLT led to a significant increase in GMV within the left amygdala. However, the expected increase in GMV within the medial prefrontal cortex was not found. Together, these findings suggest that BLT plays an important role in normalizing amygdala volume in PTSD, perhaps *via* enhanced sleep, circadian alignment, or direct stimulation that affects amygdala responses. Recent findings suggest that light exposure suppresses acute responses within the amygdala but may increase the functional connectivity between the amygdala and prefrontal cortex (Alkozei et al., 2021; Mcglashan et al., 2021; Killgore et al., 2022). Thus, repeated exposures to BLT may lead to altered patterns of activity within the amygdala that facilitate volumetric increases. On the other hand, we did not find the expected increase in medial prefrontal cortex with BLT compared to ALT. Since the medial prefrontal cortex is critical to the assessment and understanding of emotions, and the ability to regulate emotional responses, our findings suggest that most of the effect of BLT on emotion may involve bottom-up changes to the emotion responsive regions of the amygdala rather than enhancement of top-down regulatory regions of the prefrontal cortex. This should be an area for further study.

To further explore the relevance of these volumetric changes to sleep outcomes in PTSD, we examined the associations between the changes in left amygdala GMV and sleep outcomes separately for the BLT and ALT groups. Overall, we found that increases in the volume of the left amygdala were associated with improvements in subjective sleep quality and reductions in the severity of nightmares, but these associations only held as significant for the BLT group and not the ALT group. Thus, while BLT did not lead to improvements in subjective sleep quality compared to placebo overall, within the BLT group specifically, greater increases in amygdala volume were clearly associated with improvements in self-reported sleep outcomes. In other words, the associations are complex and improvement in subjective sleep appears to be related to the changes in left amygdala volume that are enhanced by BLT. Conversely, no associations were generally found between amygdala volume and objective sleep outcomes as assessed by wrist actigraphy. The only exception to this was a general association between greater increases in WASO that corresponded to increases in left amygdala volume. However, as pointed out above, WASO scores were also associated with greater time in bed, which could account for part of the association.

Together, the findings suggest that daily BLT increased time in bed, total sleep time, and left amygdala volume relative to ALT, and that within the BLT group only, greater increases in amygdala volume were associated with subjective improvements in sleep quality and nightmares. Of course, these findings should be interpreted in the context of several limitations. First, we analyzed the data using a per-protocol approach as well as a more conservative intent-to-treat approach, and found that TST was significantly improved when only those who completed the project were analyzed. However, when missing values were imputed and the larger sample was analyzed with an intent-to-treat approach, these findings were no longer significant. This suggests that the effects of BLT on TST may be a best-case scenario and may not be observed in real-life trials when participants may or may not reliably complete treatment. Further work will be needed to clarify these outcomes. Second, the sleep patterns of the participants were quite variable, which led to significant difficulties in scoring the actigraphic sleep data. To minimize the error associated with frequent daytime naps, we chose to include only data from the nocturnal sleep periods. Consequently, it is possible that different outcomes would emerge if multiple brief naps were also incorporated into the findings. Third, wrist actigraphy only provides a proxy for sleep based on activity measurements. While actigraphy correlates well with the gold standard of polysomnography, future work would benefit from full polysomnographic assessments of sleep. Fourth, the samples were smaller than initially planned due to institutional and funding agency requirements to discontinue data collection at the outset of the COVID-19 pandemic, so the modest size of the groups may have limited statistical power to detect some effects. Future research with larger sample sizes will be necessary and important to replicate and extend these findings. Fifth, the sample of participants with PTSD was quite heterogeneous with regard to their trauma histories. This made it impossible to validly compare subgroups based on trauma type. It is likely that the findings would be more telling in homogeneous groups that focused on a specific trauma exposure type (e.g., combat; sexual trauma; assault; etc.). Sixth, there was a trend toward group differences in many of the outcome measures at baseline, suggesting that the randomization process may not have completely equated the groups prior to light treatment. It is possible that some interactions may have been driven by simple regression to the mean. Alternatively, it is possible that the baseline differences may obscure even larger effects of treatment that would have been observed had the groups been equated at baseline. Finally, it is important to consider that voxel based morphometry (VBM) techniques provide only limited information about brain volume. Future work may benefit by incorporating additional anatomical approaches for gray matter (e.g., Freesurfer; metrics for gyrification, cortical thickness) or white matter integrity (e.g., diffusion tensor imaging). Nonetheless, we believe that with appropriate consideration of these limitations, the present findings demonstrate that blue-wavelength light treatment approaches may provide adjunctive non-pharmacologic intervention options that could be beneficial for enhancing some aspects of sleep and facilitating changes in critical emotional brain systems that contribute to recovery from PTSD.

## Data availability statement

The raw data supporting the conclusions of this article will be made available by the authors, without undue reservation.

## Ethics statement

The studies involving human participants were reviewed and approved by University of Arizona Institutional Review Board. The patients/participants provided their written informed consent to participate in this study.

## Author contributions

WK designed the study, obtained the funding, oversaw the collection and archiving of the data, conducted the primary statistical analyses, wrote the initial draft of the manuscript, and revised the manuscript. JV assisted in data collection, archiving of the data, preprocessing of the neuroimaging data, and contributed to draft revisions of the manuscript. ND assisted in archiving and preprocessing of the data, management of lab resources and personnel, and contributed to draft revisions of the manuscript. All authors contributed to the article and approved the submitted version.

## Footnotes

1
http://www.neuro.uni-jena.de/cat/

2
http://www.fil.ion.ucl.ac.uk/spm/software/spm12/
